# Looking at maternal health of Asháninka communities from the conceptual framework of the accessibility of care

**DOI:** 10.1186/s12939-023-01943-1

**Published:** 2023-08-14

**Authors:** Angel Oswaldo Lazo-Gonzales, Tania Sarmiento-Casavilca, Olga Elena Espinosa-Henao, Maria Guadalupe Ruelas-González, Jacqueline Elizabeth Alcalde-Rabanal

**Affiliations:** 1 Universidad Peruana Cayetano Heredia-Perú, Av. Honorio Delgado 430, 15102 San Martín de Porres, , Perú; 2 Instituto Nacional de Salud Pública de México, Av Universidad 655. CP 62100. Cuernavaca, Morelos, México

**Keywords:** Accessibility to Health Services, Maternal health, Health of indigenous populations

## Abstract

**Background:**

Peru is one of the 20 countries that has significantly reduced maternal mortality before the pandemic due to implementing policies to strengthen maternal health care, mainly in rural areas with greater poverty. However, the implementation of these policies has been different across the territory; such is the case of the indigenous communities of the Peruvian Amazon that are characterized by the inaccessibility of their territory and continue to face severe problems in accessing maternity care in health services.

**Objective:**

Analyze the main dimensions of accessibility for maternal care in public health services for women of the Asháninka community of Peru between 2016 and 2018.

**Methods:**

Qualitative research was carried out in the Asháninka community of the Tambo River. Key informants involved in maternal health care were selected, and 60 in-depth interviews were conducted that explored geographical, financial, cultural, and organizational accessibility. The interviews were recorded and transcribed into a word processor; then, a content analysis was performed to classify the texts according to the dimensions of specified accessibility.

**Results:**

Geographical accessibility: health units in the territory do not have the resolution capacity to attend maternal health problems. Financial accessibility: the programs implemented by the government have not been able to finance the indirect costs of care, such as transportation, which has high costs that a family cannot afford, given their subsistence economy. Cultural: there are efforts for cultural adaptation of maternal care, but its implementation needs to be improved, and the community cannot recognize it due to the lack of continuity of the model and the high personnel turnover. Organizational: health units are characterized by insufficient human resources, supplies, and medicines that fail to offer continuous and quality care.

**Conclusions:**

The poor geographical, financial, cultural, and organizational accessibility that women from the Asháninka community face for maternal care in public health services are evident. So, the Peruvian government must review the implementation processes of its models of care and maternal health programs in these communities and propose strategies to improve the coverage, quality and continuity of maternal care.

## Background

Peru is one of the 20 countries that has significantly reduced maternal mortality; it went from 185 maternal deaths x 100,000 NV in 2000, 55.8 maternal deaths in 2019 [1] previous the COVID pandemic, and during the pandemic this rate increased up to 88.2 in 2021 [2]This pre-pandemic achievement was the result of implementing policies to strengthen maternal health care, focusing on antenatal care in rural areas with greater poverty and high maternal mortality rates [3]. Institutional childbirth increased thanks to the promotion of waiting houses for mothers, intercultural care, and vertical birth. At the same time, the problem-solving capacity and quality of care were improved, and public health insurance (Seguro Integral de Salud in Spanish) was introduced, which finances maternal care specially for poorest people [4].

In 2017, 97.5% of pregnant women (99% urban, 92% rural) received ante*natal care* from qualified health personnel, and 88.9% received six or more antenatal consultations (90.5% urban, 84% rural). However, living in the mountains (OR = 0.85; 95% CI: 0.80–0.91), living in rural areas (OR = 0.94; 95% CI: 0.89–0.99) and belonging to a native ethnic group (OR = 0.72; 95% CI: 0.66–0.79), was associated with a lower probability of receiving a lower quality antenatal visit [5]. In the same year, 93% of deliveries were institutional (97.7% urban, 79.1% rural), being the Selva 80%) the region with the lowest coverage. Regarding the control of the immediate puerperium, 79.2% was achieved at the national level (80.4% urban, 75.6% rural), la Selva having the lowest coverage (75.3%) [6].

In Peru, around 2,703 native communities belong to 55 indigenous peoples, representing around 25% of the national population [7]. The Asháninka people are the most numerous; they are settled in the Peruvian Amazon [8]and represent about 20% of the country’s indigenous population. The global fertility rate was 4.2 times higher in native communities than in the rest of the population, and antenatal care coverage reached 60% and institutional delivery 14% [9]Only 33% of the communities have a low-complexity health facility, and 1.2% have hospitalization [8].

The picture presented in indigenous communities calls for evidence of women’s access to maternal care. For a proper understanding, we will define *accessibility* to health care as “the degree of adjustment between the characteristics and health needs of the population and the resources available for health care [10], meaning, pregnant women may receive health care when necessary[11–13]. Its field of study covers seeking and receiving care in which differentials attributed to obstacles, impediments, and difficulties or facilitators for people who require specific care are observed.

Two typologies are the most studied in the analysis of accessibility; the first analyzes the dimensions of availability, accessibility, and acceptability of health services [13, 14]. Availability is adequate when health services exist in a territory to sufficiently serve the population, are accessible if people have the necessary resources to use them, and are acceptable if they respond to the needs, expectations, and values of the beneficiaries. The second typology of accessibility analyzes the dimensions: geographical, financial, cultural, and organizational [15]. Geographical accessibility is analyzed based on the distances between the beneficiaries and the services and the possibility of covering this route with available communication and transportation [16]. Financial accessibility refers to the financial capacity of people to cover the cost of transportation, consultation, medicines, studies, and other procedures related to health care [17]. Cultural accessibility focuses on the habits, customs, and beliefs of the population around it´s health event and the differences between the professionals and the organization of the service, on one hand, and the beneficiaries, on the other. Administrative-organizational accessibility refers to institutional processes related to health care [18].

Under this conceptual framework, barriers to health care access have been identified and are conceived as factors that stand between users and health services, limiting the possibility of achieving effective and quality health care. Geographical barriers related to the location of the service, distance, time, and transportation have been identified. Financial barriers are linked to direct and indirect costs for care. Organizational barriers are linked to waiting time, timeliness of care, continuity, and adherence to treatment. Furthermore, the individual-type barriers are linked to sociocultural, religious, and behavioral aspects [10, 15].

It has been documented that the social groups with the most significant social vulnerability face barriers that impede access to health services [10]. In the case of Peru, the indigenous population is a socially disadvantaged group marked by the geographical inaccessibility of the territory where they live and by the limited development and connectivity of its people. Therefore, it is necessary to analyze and document from a holistic perspective the accessibility for health care in this population and particularly in maternal health [15, 19]. This article aims to analyze the main dimensions of accessibility for maternal care in public health services for women from the Asháninka community of Peru between 2016 2018 to generate evidence to support the implementation of strategies to improve access to maternal care and help achieve universal health coverage.

## Methods

An exploratory qualitative study was carried out between 2016 and 2018 in the Asháninca community in Satipo, department of Junín, Peru. Key actors involved in maternal health care for the Asháninka community were identified at the national and regional. to whom the scope of the study was explained in order to support its development at local level. In the territory selected for the study, coordination was carried out with the organization of the Asháninka Central of Río Tambo. In each community, a communal assembly was organized in the Asháninka language to present the research team, the objective of the study, and the methodology; also, authorization was requested for its execution.

The communities included in the study were selected for convenience, considering that (1) they belonged to the Asháninka community and (2) they were located on the banks of the Tambo River. We explore accessibility, the geographical, financial, cultural, and organizational accessibility, whose definitions are presented in Table 1. .

**Table 1:** Dimensions of accessibility explored for the care of indigenous pregnant women

DIMENSION	Definition	Analysis category	Operational definition
**Geographical accessibility**	Possibility of pregnant woman to arrive at a health facility with a resolution capacity to address her maternal health needs or problems.	Time/distance	Time it takes for the pregnant woman to arrive at a health facility to attend a maternal health event.
		Means of transportation	Most common means of transportation used in the area.
**Financial accessibility**	Possibility of the pregnant woman or her family to cover the costs demanded by the use of maternal health services.	Financial Resources	Availability of financial resources in real-time to cover the costs of transportation, accommodation, food, and clothing for the pregnant woman and the newborn.
		Strategies to subsidize expenses	Strategies to provide accommodation and temporary food to pregnant women and their close companions in order to attend a maternal health event.
**Cultural Accessibility**	Disposition of the pregnant woman and her relatives, as well as maternal health care providers, to establish a horizontal, respectful, and effective dialogue in care.	Language difference	Ability to establish effective communication between the indigenous pregnant woman and the health service provider.
		Differences in cultural conception	Differences between the conceptions, knowledge, and practices of the native pregnant woman and the care providers regarding maternal health.
		Establishment of empathy and trust	Establishment of empathic and permanent interpersonal links between native pregnant women and health care providers.
		Respectful Treatment	Equal, horizontal, and respectful treatment that the native pregnant woman or her companions receive when demanding maternal health care.
**Organizational accessibility**	Operating conditions of health services to facilitate maternal health care.	Care Regulations	Norms, procedures, and standards of care that incorporate maternal health care options with an intercultural approach.
		Resources and infrastructure	Installed capacity in terms of infrastructure and sufficiency of resources that facilitate maternal health care.
		Care processes	Organizational arrangements and procedures to facilitate maternal health care for native pregnant women.
		Culturally relevant technology	Availability of equipment or instruments for maternal health care with an intercultural and socially relevant approach.

The informants came from the study area and were pregnant women, community agents and health personnel. The criteria for they selection are described below:

- Pregnant women: between 14 and 35 years of age who were pregnant or had childbirth during 2017. Women who lived in populated centers and adjoining towns were included.
- Community agents: traditional midwives and community leaders in charge of the Health Committee who coordinate health actions with the health units and the community.
- Health personnel: responsible of the organization and maternal health provision in clinics at local level. (Ministry of health)
- Personnel in charge of maternal health programs in the Municipality of Río Tambo.

We applied In-depth interviews that explored the dimensions and categories of accessibility described in Table 1. The interview was previously piloted on five pregnant women to make suitable adjustments so that the questions were understood correctly. The research team included an indigenous translator with experience in maternal health care and the traditional midwife in the communities where she existed. Before the interview, the purpose of the study was explained to each woman, the methodology to be followed, voluntary participation and withdrawal at the time they decided, and the confidentiality of the information. Only those women who freely agreed to participate in the interview and gave their written consent were interviewed. The interviews were conducted in the indigenous language, in each woman’s home, at the time agreed with the interviewees, ensuring privacy. All the interviews were recorded and transcribed into a word processor. As a control of the transcription fidelity, interviews were randomly selected to compare the transcription with the audio.

For the dimension of organizational accessibility, in addition to the interview, a documentary review of the regulations, technical documents, and guidelines on which maternal health care is based was carried out.

A content analysis reading line by line was carried out, and the texts were classified according to dimensions and categories of analysis. The information was organized in matrices, where common and contrasting aspects of the testimonies were described in order to identify trends and conflicting aspects regarding accessibility.

The study protocol was approved by the Research Ethics Committee of the *Universidad Peruana Cayetano Heredia* in May 2017 with resolution 281-09-17.

## Results

A total of 60 interviews were conducted, of which 58% (n = 35) were pregnant women, 15% (n = 11) community leaders and agents, 8% (n = 5) health personnel, and 15% (n = 9) public officials (health and municipal workers). Whose characteristics are described in Table 2.

**Table 2:** Characteristics of key informants interviewed

Type of informant	n	Description of the participants
**Pregnant**	35	between 14–35 years old, the majority (n = 33) with a stable partner and had their first pregnancy before the age of 18 (n = 28), almost all (n = 32) had received antenatal care, and close to half (n = 32) = 16) had an institutional delivery.
**Community health agents**	02	Traditional female midwives ( 35 and 49 years old) .
**Community leaders**	09	President of the Central Asháninka organization and eight members of the Community Board of Directors, all were men, bilingual between 31 and 55 years of age.
**Health personnel responsible of the provision**	05	Three male nursing technicians, and two female midwifes, all with more than five years working in health facilities of native communities. Between 24–56 years old.
**Health personnel responsible of the organization of maternal health programs**	06	Managers of two health micro-networks, Coordinator of the Sexual and Reproductive Health Strategy, Coordinator of Health Programs of the Satipo Hospital, Coordinator of Reproductive Health of DIRESA, and the Coordinator of Indigenous People
**Municipal personnel of Rio Tambo**	03	Two persons in charge of Social Development, and an employee of the Rio Tambo Municipal Shelter, their ages ranged between 28 and 37 years
**Total**	**60**	

### Geographical accessibility

The native population in Satipo is characterized by living in small and dispersed communities, mainly located on the riverbanks. The primary means of transport is by the river, and characterized by low demand and supply (only once a day), high operating costs, and minimal safety measures. The time to commute from the native communities to the health center (HC) in Puerto Ocopa, where maternal health can be provided is between 3 and 8 h minimum, and transportation costs range from 30 to 70 US dollars (Table 3). This HC receives referrals from the health posts in the Tambo River basin and, in case of emergency this unit refers pregnant women to the Satipo Hospital, which is 1 h away by land, and transportation is frequent during the day.

**Table 3:** Travel time and costs of care from the communities to the Puerto Ocopa Health Center

Health Facility	Time-distance (hours)	Transportation	Transportation’s approximate costs
Ps. Los Ángeles de Shima	8.00	River/Land	**US $21**
Ps. Boca Chembo	7.00	River	**US $21**
Ps. Impaniquiari	6.00	River	**US $21**
Ps. Santa Rosita de Shirintiari	6.00	River	**US $18**
Ps. Shevoja	5.30	River	**US $18**
Cs. Betania	5.30	River	**US $18**
Ps. Capitiri	5.00	River	**US $15**
Ps. San Francisco de Cushireni	4.30	River	**US $13**
Cs. Poyeni	4.00	River	**US $12**
Cs. Oviri	3.30	River	**US $ 9**
Ps. San Miguel de Otica	3.00	River	**US $ 9**

Source: Amazonian Indigenous Peoples Management - Municipality of Río Tambo

Health facilities in this area are distributed in the river basins (Tambo, Ene, and Perené). The conditions of the Amazonian geography make it difficult for pregnant women from communities far from the riverbanks to access care, and the cost of transportation is high.

*… the labor pains began at night, coming from the farm at night is very difficult (…) There was no “Peque”*^1^*for her to come, that is why she gave birth there. (G1****Otica****)*

.. *we have pregnant women from 8 to 13 hours away; they cannot reach the health facility, nor can the staff reach the community. They are cared traditional midwives or by the mother” (****FM 01 RT)***

### Financial accessibility

#### Financial resources

The Asháninka people’s main subsistence activities are hunting and gathering fruits for self-consumption and exchange (barter). It does not have cumulative purposes; this economy remains unchanged, which is why the families’ income is meager. The chances of getting a job are poor due to the lack of investment of the companies in this area

*“… here you can see extreme poverty; The level of poverty is so great that many people prefer not to refer their patients because the expense for care is so much that they prefer their death at home.” (FS 01 PO)*.

These families use the little cash that they get for buying their food and therefore do not have cash to pay for their health expenses. Financial difficulty for women’s health care aggravates when supplementary examinations are required because they must commute to a distant facility.

... *he sent me to Puerto Ocopa to have an ultrasound done... they were telling me to go, but I didn’t have (money) to go. (G20-Otica)*

### Strategies to subsidize expenses

Maternity homes are one of the main strategies implemented to face geographic and financial barriers to care for Asháninka women. However, at the time of the study, the maternity home of the Puerto Ocopa Health Center was not working, since this space in the health unit was used as a warehouse; it was closed due to the lack of resources from public health insurance for its maintenance. Some pregnant women interviewed were unaware of the existence of the maternal home.

*Anytime I used the house for mothers because it only works at the begging four years ago. I heard it is not working for the lack of budget and I don’t have money .“ (DC_Otica 06)*

The JUNTOS program offers families with school-age children and pregnant women from poorer communities a subsidy of 100 PEN (approximately US$30) per month to encourage adherence to antenatal care. In the native communities of the Rio Tambo district, coverage of 87.1% of this program has been estimated [8]. The amount that families receive is very little, not even enough to cover the cost of transport. This precariousness induces the woman and her family not to go to the health care center, even when the woman has risk factors.

*Regarding the JUNTOS Program… “Of course, since they are paid, as it is their responsibility, they come to tell you: “I am pregnant, miss, and I want to do my checkup.“ (PS 01 PO)*

*I am beneficiary of the JUNTOS Program, the money that I receive is very good for mi family but the amount is not enough for paying peque of me and my husband to go to the clinic.“ (DC_Otica 25)*

It is common for communities, through their authorities, to participate in the transfer of obstetric emergencies and finance the transportation expenses for the pregnant woman and a companion to a health center. Even so, there are difficulties for the return; the companions or relatives must pay.

*“We have small funds, and depending on the case, we support transportation. Now, in case there are no funds, we have to look for or lend fuel for the peque so that they can move it immediately” (DC 05- Otica)*

### Cultural accessibility

#### Language difference

Undoubtedly, one of the main barriers to maternal care is the language between the Asháninka communities and the health personnel. The health personnel who work in these communities do not speak the local language, which is why there are communication problems.

*“This is what limits (the language) in the Puerto Ocopa Health Center because the professional who attends does not know the AsháninKa language” ( G 023 Otica )*

*… “An Asháninka person is humble, sometimes they don’t understand Spanish: Worse! That’s worse! They leave a person (Ashaninka woman) there (health center) until someone (health peronnel) who understands their language comes” (FM 02 RT)*

### Differences in cultural conception

The lifestyle, perceptions and health customs in Amazonian communities and health personnel are sometimes confrontational. Health personnel recognizes difficulties in understanding and establishing harmonious relationships due to the conceptions, beliefs, symbols, and rites around women’s health alien to them.

*“(...)... the vast majority of us come abroad, we have a different experience, right? We have other customs and other beliefs, and when one arrives as if it shocks them, it is a little more difficult to adapt... “(PS 02 PO)*

*“(...)...the personnel at the health center judge me as I am dressed, they tell me to rest, but I don’t do it because the child will be lazy. G 07 PO*

During childbirth in native communities, the use of steam is frequent since hotness is associated with flexibility; that is, it is perceived that the hot hip will be more flexible for childbirth. It is common to ingest infusions of plants and overcoats during childbirth. However, the community is afraid to share these practices because they perceive that their traditional knowledge and practices are despised.

*“My aunt has steamed me. She has rubbed me; then the head (of the baby) goes down quickly” (G 09 PO).*

*“Before going to the post (Health Center), I took the Piripiri. that’s why (the baby) has come out. If she had not taken it, the baby would have died” (G 04 PO).*

In a pregnant woman with a partner, the man takes control of the sexual and reproductive life of “his wife”. He decides if ¨the pregnant woman goes to the health center or not. His support is fundamental since most of the Asháninka men speak Spanish and the women do not.

*“It’s more than anything her culture, her way of thinking, the machismo of her husband and family members. That’s what they say… “If my mother has given birth at home, she has given birth to 8 or 10 children; why can’t my wife give birth at home? She’s young”; so, she has to give birth at home” (PS 01 PO)*

*”Their husbands do not accompany them to the health center; therefore, I have to accompany them to help her with the translation because otherwise, she does not understand what the nurse says, so I am worried about taking responsibility for the family…” (DC 01 OTICA).*

The mother in the Asháninka culture is a source of knowledge about sexuality and reproduction. She is responsible for teaching her daughters about care during pregnancy, childbirth, and postpartum concerning food, care, and “how to give birth” when visiting the midwife, among others. Pregnant women, especially the first time or those with complications, go to a woman in the family with previous experience.

*“(The baby) was crossed, it hurts. When I was five months old, they told me to fix it, and it didn’t hurt anymore, so they rubbed me with menthol, they also shook me” (G 02 PO)*

If the family member or midwife cannot resolve the problem, they request the presence of health personnel at the pregnant woman’s home. Nevertheless, he should not touch or see the genitals of the pregnant woman since exhibiting her genitals’ during childbirth is a source of great shame. The most remote communities reject institutional childbirth, conceive childbirth as a private event and choose to give birth alone in their homes. In Otica, practically all pregnant women give birth in their homes surrounded by extensive social support and are proud of it.

*“…. They don’t want them to see her parts (genitals) when giving birth. They oppose opening the legs, so the staff have to do it by force (open the legs of the pregnant woman)” (DC 02 OTICA)*

*… some women have their check-ups done… but when they are close to giving birth, they go to the mountains; they suddenly cling to coconut or bamboo trees and give birth alone at home or on the farm (PS 01 PO)*

### Establishment of empathy and trust

As part of the intercultural process for childbirth care, health personnel have accepted local practices related to deliveries like: heat and the use of infusions during childbirth, soft massages,

slightly accommodate the child, position that the woman prefers for deliver and use of extra blankets during labour

*”...I have also had to get involved with them (pregnant women). Did you work with Piripiri? Yes, I have; It has made it easier for me to get closer to women…” (PS 01 PO)*

*“ In the clinic midwife delivers my baby in the position of my choice, this is very good, I hold on to something and push standing upright. (RT_06)*.

In the event of a referral to the Satipo Hospital, the pregnant woman and her family face the hostile climate of the Hospital, where “no one gives reason for anything.“ They feel discriminated against due to their ethnicity and poverty; they are treated as “dirty or dirty people,“ and in some cases, they face explicit mistreatment. The Hospital does not have bilingual personnel, which generates greater cultural disagreement when trying to understand administrative procedures that are foreign to them, such as lines, allocation of quotas, tickets, and registration of formats.

*“…in the hospital, they report that they are mistreated, that they do not understand them, that they are not allowed to do anything, but here (Health Center), we have them walk with the whole family, but for the delivery there, only she enters, and We rarely allow the family to enter because sometimes they don’t understand what we do” (G 01 Otica).*

A pregnant woman does not find social support in the city, she and her relatives find it inhospitable. She also faces communication limitations, scarce resources, ignorance of city customs, and a discriminatory attitude towards them from its habitants.

*“Because (they) don’t have an economy. Others for social reasons: they do not have adequate communication. Little do they make themselves understood” (FM02 RT)*

### Respectful treatment

Native pregnant women have come to fear care in health centers. Women perceive that they are not respected because health personnel do things that they do not approve like touch and look at their genitals. They also acknowledge that health personnel treats them with violence.

*“There are some (people) who tell me they (health personnel) check your parts (...) that’s why they are afraid to go to the clinic " (G 06 PO)*

*”But some ladies tell you... I had a friend who also had three babies in a row, the midwife told her: have you had that many babies?“ (G 04 PO)*

Abuse towards health personnel form pregnant woman is also recognized, which restricts the possibility of dialogue between pregnant Asháninkas and health personnel.

*“They (pregnant women) came to the service, grabbed their card, and threw it at the table, and I said: Why are they mistreating me like this? What is happening, or they don’t like me at all, or I don’t know” (PS 01 OP)*

However, health professionals highlight that they are looking for new ways to relate to the native pregnant woman, such as preferential care and acceptance of cultural patterns.

*“... so, I tried to get involved with them and accept what is good for them, and show them that I accept them, right? and you don’t say anything (...), and they come. (PS 01 PO)*

### Organizational accessibility

#### Care regulations

The documents regulating health care focus on the standardization of care processes as a strategy to guarantee and ensure the quality of the implemented processes. In none of them considers strategies to respond according the characteristics of the population and the context specially in excluded communities and communities with significant cultural differences.To strengthen the coverage of maternal health care, the Public Health Insurance (SIS for its initials in Spanish) includes free care guidelines for maternal health and pregnant women transfer.

*“The Insurance reimburses all the activities we do. If we take advantage of all the activities an arriving patient does, the SIS will reimburse you as long as we put all the activities in the attention form.“ (FS 01 HMH)*

There are no clear communication mechanisms between the community and the health services to organize the women transfer in case of emergency. Although this should be covered by the SIS, in practice the family must organize the transfer and cover its costs.

“…..they say that the SIS pays to take us to the hospital if we have bleeding, but we don’t know who to contact for help. That is why, if we have money, we go and if we don’t, it’s better to stay here (her home). *G 09 PO)*

According to the regulations, for reimbursement health centers must record information about the care given in standardized forms. Problems with reimbursement emerged due to the resistance of the personnel to properly record the information required.

*“(…) there are staff, graduates, who say: “I don’t work for the SIS”; It is not that you work for the SIS, you work for the State, you work for the population, you are here to serve the population, do you understand me?“ (FS 01 HMH)*

According to the norm, only the Satipo Hospital would be authorized to attend deliveries, but this is inaccessible to most of the Asháninka population, even more for pregnant women. Units categorized as I-3 Health Centers have minimum conditions for the care of “imminent delivery”; one is the Puerto Ocopa Health Center.

### Resources and infrastructure

Some public initiatives to strengthen maternal care are highly valued, such as the Municipal Incentive Program and the Performance Incentive Fund (FED for its initials in Spanish); however, inadequate management and misuse of resources are reported in this program. The availability of supplies and materials for maternal care are not constant since the program face logistical problems for delivering supplies and materials, so that health personnel must assume the cost of transporting without receiving reimbursement,

*“We don’t have the FED; a couple of years ago, the municipality had spent the funds on something else, then the FED was lost... so they already supply us with the inputs we need” (PS 02 PO)*

The Municipality of Río Tambo has formed a network of complementary health services to the public network, comprised of 14 health posts and 15 community first-aid kits located in the most inaccessible communities. However, the efforts are insufficient, and the Satipo Health Network reported inadequate coordination with units of Ministry of health and technical and logistical support.

The Ministry of Health units in this area are characterized by scarce equipment and equipment that require maintenance e.g The ultrasound machine is broken.*“ The device to take pictures of my baby has not been working for a long time, I can’t go anywhere else to take the picture, I don’t have any money” (G21 03 PO)*

The Ocopa Clinic has medical personnel, professional midwives, and dentists for prenatal care, childbirth, family planning, and adolescent health care. These personnel cannot cover post-call breaks, vacations, training, and leaves of absence.

The Ótica Health Post has a health technician and a nurse or midwife who perform their social service for one year, after which they return to their original place. Transportation for fluvial mobilization is inoperative

*“We have an engine, but we don’t have the “peque”*^2^. *With the community, we made a wooden canoe, but it was damaged, and it doesn’t work either. We have taken steps with the municipality, and they told us: “Yes, yes, yes, don’t worry” I will support you with the little one, but there are no results” (PS 03 PO)*

*(An aluminum boat) “It would be much better, and that would make any emergency easier for us” (PS 03 PO)*.

### Care processes

Formally there are no delivery services available in the entire Rio Tambo basin. The HC Puerto Ocopa offers maternal healthcare (antenatal care, deliveries, postpartum care, family planning methods, self-education in health) and Maternal Waiting House; however, at the time of the study, this space was being used as a warehouse. This clinic receives referrals from women with risk factors in pregnancy of the Health Posts of the Rio Tambo basin and refers to the Satipo Hospital women with pregnancy complications that they can’t handle.

*“the clinic provides care for simple health problems, if there are severe complications in which our life or the child’s life is at risk, we are sent to the Satipo Hospital*” *(G 17PO).*

The health post only performs antenatal care, family planning methods, self-care education and care for imminent delivery. The health services do not guarantee the transfer of the pregnant woman in risk cases.

...*the “Maternity Homes” have no solvency, so who will support them? Because the SIS does not support all the houses since they are not recognized…” (TS 02 PO)*

In case of a delivery complication, referral to a Health Center or Hospital is chosen, for which the community authorities organize the transfer of the pregnant women.

*“After four months (I realized) (…) (my mother) took me to the post” (G 06PO).*

*“I went to the post with my grandmother” (G 05PO).*

### Culturally relevant technology

The prenatal care model follows national guidelines. Up to now no national or local effort has been made to adapt prenatal care with an intercultural perspective. In other words, a model that incorporates beliefs and practices of the native community without any harm to the mother and child, and that brings women closer to health services

“*The midwife tells us that we have to eat meat and chicken, but there is no money, we can only eat birds that we catch. She doesn’t put herself in our shoes. She also tells us to exercise, so that we don’t have pain in labour, but we work a lot in the fields, we think that’s enough, we don’t have any pain in labour”. (G 19PO).*.

In both health centers, health personnel have the necessary accessories (bench and support ropes) for vertical delivery care, according to current regulations. In the HC Puerto Ocopa and Otica health posts, 22% and 100% of the pregnant women opted for this mode of delivery, respectively. On the other hand, it is essential to highlight that in these units, most of the personnel are foreigners, therefore strangers to the Asháninka culture and obviously to their language.

*“When staff leave for training, they leave their service empty... there are times when Obstetrics is closed” (FS 01 PO)*

## Discussion

It is evident that despite the policies and programs implemented by the Peruvian government to improve access to health care in pre-pandemic period for the most vulnerable population, geographic, financial, cultural, and organizational barriers persist that limit maternal health care for women of the Asháninkas communities [20]. This result agrees with findings that show that population with significant social disadvantage have less access to health services [21]. In the case of the communities of the Peruvian Amazon, given their geographic, social, and economic exclusion, few studies document the poor access of this population to maternal health services [22–24]. and after the pandemic the maternal health programs in this communities could be worst.

The native communities of the Amazon are characterized by being located on the banks of rivers that are difficult to access; there are insufficient means of transportation and communication routes that do not facilitate the rapid mobilization of the population and health personnel in case of a maternal emergency [25]. The limited investment of the state in these communities is evident, resulting in low development that limits their economic and political integration and facilitate inequity and social exclusion [26]. Geographical inaccessibility has severe implications in terms of displacement, transportation costs are high, and many families, due to the poverty in which they live, cannot afford the costs [27]. This situation has effects on the use of maternal health services [28, 29, 5], which decreased during the pandemic [30], the general coverage of antenatal care was 81.5%, institutional deliveries 93% and access to modern contraceptive methods was 53% but in the forest was 77.8%, 81.6 and 55% respectively. And probably the coverage in the inaccessible areas are lower [31].

During the last decades, instead of improving the standard of living of indigenous peoples, they have put their fragile subsistence economy in crisis with a market model that excludes development proposals with an intercultural, inclusive, and ecologically sustainable approach that has accentuated poverty [32]. The poverty of the native communities constitutes one of the main barriers to access to maternal health care, given that their low purchasing power prevents them from paying a series of out-of-pocket expenses for pregnancy and childbirth care that is becoming critical if complications occur [33]. Other regional studies have similarly documented economic barriers [34].

Cultural conceptions and beliefs strongly influence maternal health care; pregnancy is an important event in the life of the Asháninka women; it marks the transition from being “ewankawoy” to being an “antaotake”, which means being prepared to be a mother [35]. In native communities, pregnancy and childbirth are conceived as natural biological processes; therefore, these events must be accompanied by the family and managed in the domestic environment. This model conflicts with the biomedical one and represents a barrier that impedes provide culturally appropriate maternity care by health personnel [36] and according of the social context of the Asháninka community [37, 38] Maternity homes strategy had a successful response in Andean communities, but they are perceiving by Amazonian communities as spaces where their beliefs and customs are threatened because childbirth occurs in an environment that is foreign to them and far from their family [39].

The poor understanding of the use of traditional medicine in maternal care by health personnel generates prejudices that can result in an offensive or inappropriate assessment of the practices and customs of native communities [40]. This cultural disagreement subordinates native customs and ways of life and generates attitudes of resistance, fear, and distrust of the Asháninka community towards health workers, consequently affecting the search for care [27, 26]. Native women living in jungle regions have less access to quality antenatal care, in addition to less probability of delivery care by qualified personnel [41, 42] [5].

Regarding organizational accessibility, the regulations for maternal care in Peru are designed to respond homogeneously in a heterogeneous country. Hence, the Asháninka population perceives that the care offered in health services is far from their culture and customs. The JUNTOS Program has not achieved the coverage and opportunity in antenatal care, as well as the coverage in deliveries [2], that may be even lowerin non-Spanish-speaking native communities that are object of discrimination, mistreatment, and deficient care in health services [43, 44][45]. The Municipal program had no results since It do not have continuous implementation, face problems of supplies and materials and its coordination with local health services is scares. Its objective is only to fulfil its political commitments without any real interest in results. On the other hand, according to the norm of “categorization” of health center by obstetric function, deliveries should not be attended in this units.

However, these health units are the most accessible to these communities, where there is a shortage of qualified personnel for maternal care, medicines, and equipment. Most of them work with social service personnel, their permanence is one year, so it is necessary to offer incentives and adequate working conditions that allow them to remain in these areas of difficult access [27, 46]. As part of the incentives to assure the retention of health workers in other countries was an additional payment [47–49] appropriate housing facilities [50]; educational incentives and developing of the professional carriers to have better position in public health service in the future. In the rural areas the majority of health professionals require continuous full working hours and a rest period, i.e. 15 days of continuous work by 15 days off. They argue that this schedule allows them to organize their work and their personal life. The Asháninka woman not only faces a lack of personnel, but she must also deal with personnel who do not speak the indigenous language and lack knowledge and skills for intercultural care [47]. It is no coincidence that institutional delivery coverage in Peruvian Amazonian communities is 4.5 times lower than the national average [51].

## Conclusion

The results show the poor geographical, financial, cultural, and organizational accessibility that face women from the Asháninka community to receive maternal care in public health services in Peru. These findings highlight the disadvantage of indigenous communities to receive care in health services and reveal that maternal health policies for vulnerable groups, as well as models of care, have not been able to include this population. Therefore, achieving universal health coverage for the indigenous population is still a long way off. Consequently, the Peruvian government must review the implementation processes of its maternal programs, as well as the models and strategies implemented to improve coverage, quality and continuity of care in this communities. On the other hand, it is necessary to conduct more frequent research, evaluation and monitoring of maternal programs in these population groups, in order to have evidence to support their targeting and prioritization of intervention in the public agenda, as well as the intercultural models to be implemented to achieve better health outcomes.

## Data Availability

Not applicable.

## Footnotes

1 “Peque” is the onomatopoeic name of the simple motor boat, which serves as river transport among the community. It is usually said “pequepeque” its simplification is “peque”

2 “Peque” is the onomatopoeic name of the simple motor boat, which serves as river transport among the community. It is usually said “pequepeque” its simplification is “peque”
